# Value and relevance of routine postoperative blood sample analysis after general surgery-a single centre analysis of 1198 patients

**DOI:** 10.1007/s00423-025-03726-4

**Published:** 2025-05-29

**Authors:** Dalil Ali, Claudius Juergens, Markus Unnewehr, Maximilian Schmeding

**Affiliations:** 1 Department of Surgery, Klinikum Dortmund, Beurhausstrasse 40, 44137 Dortmund, Germany; 2 Department of Medicine, Faculty of Health, Witten/Herdecke University, Alfred-Herrhausen-Straße 50, 58448 Witten, Germany; 3 Faculty of Medicine, RWTH Aachen University, Pauwelsstr. 30, 52074 Aachen, Germany; 4 Department of Respiratory Medicine, Infectious Diseases, Sleep Medicine, Allergology, St. Barbara- Klinik, Am Heessener Wald 1, 59073 Hamm, Germany

**Keywords:** Inflammation, Lab value, Postoperative contol, General surgery

## Abstract

**Background:**

Routine blood sample analysis is performed in most surgical institutions after general surgery on the first postoperative day. Substantial economical impact is thereby generated in addition to patient distress caused by venous puncture.

**Aim:**

The presented study was designed to analyse the relevance of postoperative routine blood sampling with special focus on patient safety.

**Methods:**

1198 patients undergoing minor general surgical procedures (appendectomy, cholecystectomy, groin hernia repair) at our institution were retrospectively analysed. Data was gathered and forwarded to statistical evaluation with respect to patient safety and economical impact / cost-saving potential.

**Results:**

In 5,3% of our patients there was clinical impact of postoperative blood sampling. Relevant medical treatment consequences were derived for complicated appendectomy cases only.

**Discussion:**

Our data suggests that routine postoperative blood sample analysis after minor general surgery has very little impact on the further clinical course. Patient safety is not at risk. Regarding the economical and distressing impacts of routine blood-drawing post-operatively it should be evaluated if routine sampling may be limited to special interest cases.

## Introduction

General surgery procedures of limited complexity account for a substantial proportion of in-house patient treatment in the western world. Inspite of continuous efforts to increase ambulatory surgery and postoperative care the number of in-house cases remains on a relevant level [1]. Whereas other western countries have moved to almost exclusive ambulatory treatment for procedures such as inguinal hernia repair [2], Germany has initiated this development only recently [3–5]. Other minor procedures such as appendectomy, cholecystectomy [6] or thyroid resection remain largely to be treated as in-house cases [7]. For most patients blood value analysis is performed prior to surgery, either acutely in the „emergency setting“ or electively for planned patients. This is neccessary in order to rule out unknown pathologies potentially inflicting anethesia and surgery before the procedure [8].

On the first postoperative day (POD1) blood sample analysis is performed once again as a routine tool after surgical treatment [9]. Theoretically, abnormalities in haemoglobine levels, liver and kidney function parameters and coagulation and inflammation values could be detected [10].

However, the clinical significance of this routine POD1-blood sampling is questionable [11–13]. Regarding the fact that each blood value analysis contributes to substantial economical and personal ressource impact combined with individual patient distress [14] by venous puncture it may be discussed if routine employment of POD1-analysis is useful. Continuously expanding ambulatory surgery without POD1-blood sampling further stimulates this discussion.

Although the amount of lab values analysed in POD1-blood sample analysis has already been limited to only 10–12 parameters in our institution the aspects mentioned above remain relevant.

Therefore the aim of the presented study is to analyze, if routine POD1-blood sample analysis has an impact on the further treatment course of the individual patient. It was hypothesized that other parameters such as clinical evaluation, pain scoring and patient activity may reflect a sufficiently precise picture of the patients‘ health status and blood sampling may only be performed when clinically indicated [15].

We conducted a single-centre retrospective analysis on 1200 patients treated with minor general surgery at a large communal tertiary hospital in Germany. Procedures included were appendectomy, cholecystectomy and groin hernia repair. Patients with preoperative or intraoperatively detected substantial inflammation (cholecystitis, advanced appendicitis) were ommitted from this analysis.

## Materials and methods

### Study design

This study is a retrospective two-arm controlled confirmatory observational study. Allocation to the study arms was based on the assessment of whether the postoperative laboratory values were normal or abnormal overall.

All analysis was carried out in accordance with current applicable ethical guidelines.

### Patients

Laboratory findings, patient characteristics and postoperative complications were extracted from the digital patient files in the patient information system of Dortmund Hospital and data obtained as part of quality assurance measures.

All patients receiving an appendectomy, cholecystectomy or inguinal hernia repair at the surgical clinic of Dortmund hospital between January 1, 2021 and December 31, 2022 (731 days) were examined for inclusion in the current study.

### Inclusion and exclusion criteria

All patients with the following minor visceral surgery operations were included:

- Appendectomy for appendicitis (K35.30, K36, K37, K38.9).
- Cholecystectomy (K80.20, K80.10).
- Inguinal hernia repair (K40.09, K4091, K40.20, K40.21).

Patients were excluded from the study, if they show the following, intraoperative findings:

-complicative appendicitis (K35.2, K35.31, K35.32).
- acute cholecystitis (K80.00).
- acutely incarcerated inguinal hernias (K40.40, K40.41, K40.30, K40.31).

Furthermore, all patients with further complications during the operation or deviation of the planned surgical procedure were exclude, these included:

-severe adhesions.
-monstrous hernias.

### Recorded data

- **Baseline data**: Age (in years), gender (male/female), known comorbidities (negative/positive, the patients are considerd comorbid if they have an ASA-classification III or higher).

ASA-classification (American Society of Anesthesiologists):

**Table:** 

ASA-classification	Definition
ASA I	A normal healthy patient
ASA II	A patient with mild systemic disease
ASA III	A patient with severe systemic disease
ASA IV	A patient with severe systemic disease that is a constant threat to life
ASA V	A declared brain-dead patient whose organs are being removed for donor purposes

- **Laboratory findings**: Summarized findings of pre- and postoperative laboratory values (normal / abnormal / no laboratory check) in terms of clinical relevance.

summarized laboratory values were considered “abnormal” if one or more of the laboratory parameters were found to be abnormal. For this classification, the following reference / limit values were defined here:

**Table:** 

Laboratory findings	normal	abnormal
Leukocytes	4,000–10,000/µl	> 15,000/µl
Hemoglubin	12–17 g/dl	< 10 g/dl
Bilirubin	1–1.2 mg/dl	> 2 mg/dl
Potassium	3.5–4.5 mmol/l	< 3 or > 5 mmol/l
CRP	< 5 mg/dl	> 100 mg/dl

-**Operations**: appendectomy, cholecystectomy, inguinal hernia repair (both Lichtenstein and TEP procedures).
-**Postoperative procedure**: consequences taken (yes, no) and if yes,

postoperative procedure initiated (none / one of 7 interventions):

the relevant interventions were:

- inpatient antibiotic therapie.
-laboratory check on day 3 postoperatively.
-therapeutic intervention, i.e. ERCP, blood transfusion and CT guided percutaneous drainage.
-hospital-readmission after discharge.
-intravenous potassium replacement.
-outpatient laboratory check.
-outpatient antibiotic therapy.
-length of hospitalisation (regular / deviating, defined as more than 2 days after surgery).

Postoperative complications were defined as follows:

For inguinal hernia repair: postoperative haematoma / severe pain / nerve injury / wound infection.

For cholecystectomy: bile leackage / bile duct injury / haematoma with need of drainage in operative field / wound infection.

For appendectomy: persistent (local) peritonitis with need of re-intervention / appendectomy stump insufficiency / haematoma in operative field/ wound infection.

### Target parameters

The primary endpoint was discharge without complications, i.e. without consequences (no necessary procedures), without abnormalities and within the regular length of hospitalisation.

The aim was to test the above-mentioned primary hypothesis as to whether postoperative laboratory monitoring has benefits and effects on the further course of treatment in minor visceral surgery.

### Statistical evaluation

The descriptive analysis followed the current APA guidelines: Variables at nominal scale level are presented with absolute and relative frequency, metric variables (concerns only age) as mean and standard deviation.

The hypotheses regarding the primary and secondary outcome parameters are tested using the chi-square test (X² test).

Dependencies of the target parameters on age are evaluated using a t-test.

As is usual in exploratory studies, all target parameters are tested against an uncorrected alpha = 0.05 (i.e. without Bonferroni correction). All resulting probabilities with a *p* < 0.05 are therefore not considered confirmatory, but only “probatory” (temporary) or “exploratory significant”, i.e. interesting for more precise testing in a future confirmatory study.

Two separate logistic regressions were used to compare the significance of the individual pre- and post-operative factors in relation to the two target variables of consequences (yes / no) and extended length of stay (yes / no).

The statistical program R (R-Studio, version 4.1) was used for the statistical analysis.

## Results

### Patients

**Fig. 1:**
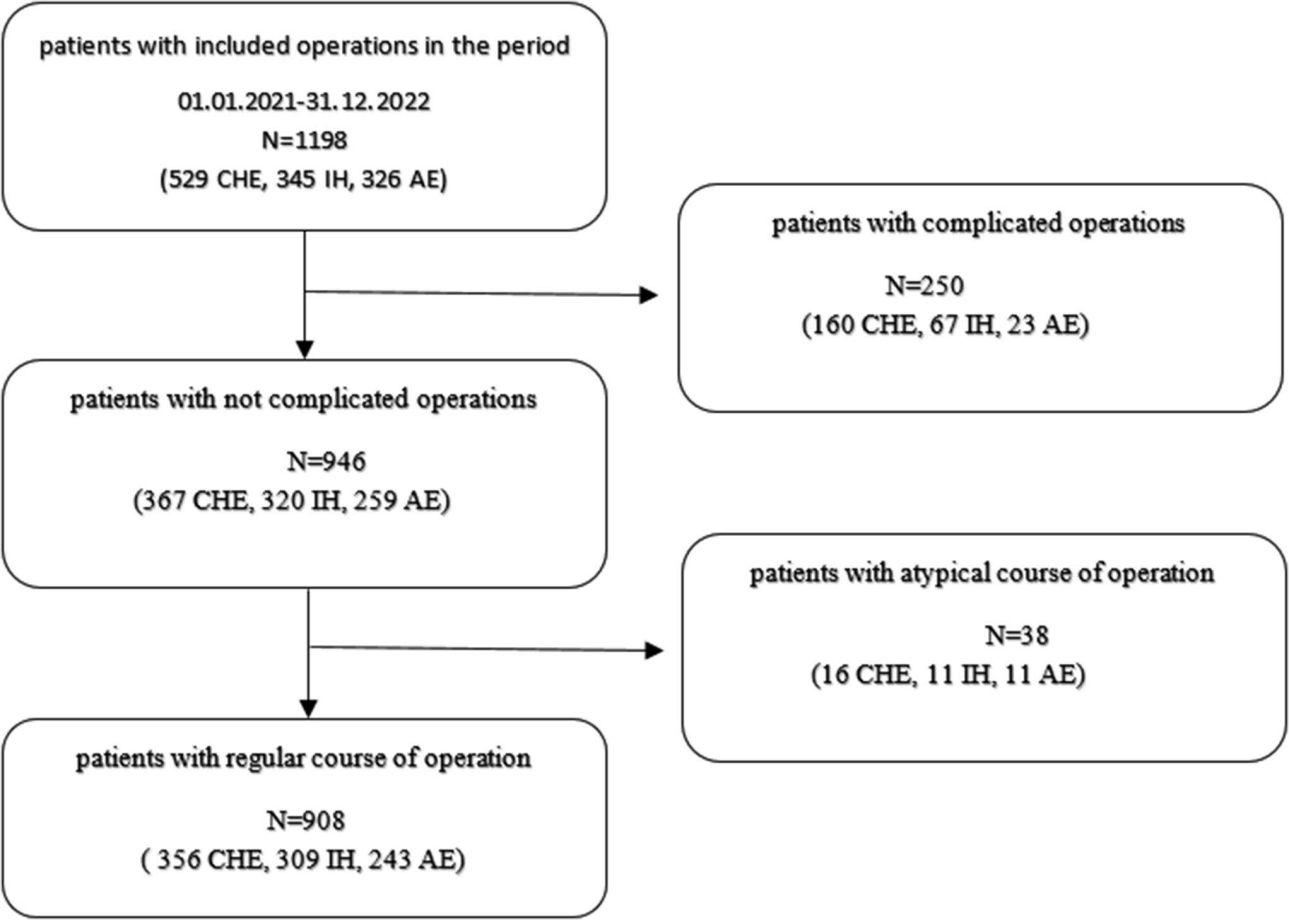
Consort Chart of this retrospective registry data study. N = absolute frequency, CHE = cholecystectomy, IH = inguinal hernia repair, AE = appendectomy

A total of *N* = 1198 patients were examined, who underwent one of the three operations: appendectomy, cholecystectomy, and inguinal hernia operations, at the Surgical Clinic of Klinikum Dortmund between January 01.01.2021, and December 31.12.2022. Of these, 250 cases with intraoperatively detected complications (i.e., acute cholecystitis, acutely incarcerated inguinal hernias, and complicated appendicitis) were excluded. Furthermore, 38 cases were excluded due to an atypical course of surgery, resulting in a final inclusion of *N* = 908 patients (Fig. 1).

**Table 1:** Descriptive results of the study population as a whole (*N* = 908 patients), as well as divided for patients with abnormal (*N* = 129) and normal (*N* = 779) postoperative laboratory results. The statistical comparison provides the parameters of the t-test (parameter age) and the Chi-squared test (all other parameters). For the parameter age, descriptive measures are given as m = mean ± sd = standard deviation, for all others in n = absolute frequencies (and relative frequencies in percent)

Postoperative laboratory values					
Parameter		All patients	Abnormal	Normal	Stat. comparison
Number		908(100.0%)	129(100.0%)	779(100.0%)	
Age	M ± SD	47.9 ± 18.98	52 ± 19.74	47.2 ± 18.7	T = 2.71, *p* = 0.007
Gender	Male Female	479 (52.8%) 429 (47.2%)	85 (65.9%) 44 (34.1%)	394 (50.6%) 385 (49.4%)	X²=10.41, *p* = 0.001 X²=10.41, *p* = 0.001
Comorbidity	ASA3 or more no comorbidity	143 (15.7%) 763 (84.0%)	30 (23.3%) 99 (76.7%)	113 (14.5%) 664 (85.2%)	X²=6.39, *p* = 0.012 X²=5.95, *p* = 0.015
Preoperative laboratory values	Abnormal normal	75 (8.3%) 832 (91.6%)	36 (27.9%) 92 (71.3%)	39 (5.0%) 740(95.0%)	X²=76.59, *p* = 0.001 X²=80.89, *p* = 0.001
Operation	Cholecystectomy inguinal hernia repair appendectomy	356 (39.2%) 309 (34.0%) 243 (26.8%)	43(33.3%) 38(29.5%) 48(37.2%)	313(40.2%) 271(34.8%) 195(25.0%)	X²=2.18, *p* = 0.140 X²=1.40, *p* = 0.207 X²=8.37, *p* = 0.004
Postoperative laboratory values	Abnormal normal no check	129(14.2%) 754(83.0%) 25(2.8%)	129(100%) 0(0.0%) 0(0.0%)	0(0.0%) 754(96.4%) 25(3.2%)	X²=908.00, *p* = 0.001 X²=736.19, *p* = 0.001 X²=4.26, *p* = 0.039
Abnormal laboratory parameter	leuokocytes or crp hemoglobin creatinin bilirubin potassium more than one	101(11.1%) 1(0.1%) 5(0.6%) 8(0.9%) 3(0.3%) 4(0.4%)	101(78.3%) 1(0.8%) 5(3.9%) 8(6.2%) 3(2.3%) 4(3.1%)	0(0.0%) 0(0.0%) 0(0.0%) 0(0.0%) 0(0.0%) 0(0.0%)	X²=686.25, *p* = 0.001 X²=6.05, *p* = 0.014 X²=30.036, *p* = 0.001 X²=48.74, *p* = 0.001 X²=18.18, *p* = 0.001 X²=24.26, *p* = 0.001
Consequence	Yes No	48(5.3%) 858(94.5%)	48(37.2%) 81(62.8%)	0 (0.0%) 777 (99.7%)	X²=306.04, *p* = 0.001 X²=290.43, *p* = 0.001
Procedure	inpatient antibiotic therapy labor check on day 3 therapeutic intevention hospital-readmission potassium replacement outpatient lab. check outpatient antibiotic therapy	27 (3.0%) 7 (0.8%) 4(0.4%) 1(0.1%) 2 (0.2%) 6 (0.7%) 1 (0.1%)	27 (20.9%) 7 (5.4%) 4 (3.1%) 1 (0.8%) 2 (1.6%) 6 (04.7%) 1 (0.8%)	0 (0.0%) 0 (0.0%) 0 (0.0%) 0 (0.0%) 0 (0.0%) 0 (0.0%) 0 (0.0%)	X²=168.04, *p* = 0.001 X²=42.60, *p* = 0.001 X²=24.26, *p* = 0.001 X²=6.05, *p* = 0.014 X²=12.10, *p* = 0.001 X²=36.47, *p* = 0.001 X²=6.05, *p* = 0.014
Duration of hospitalisation ( normal ≤ 2 days)	abnormal (> 2days) normal (≤ 2 days)	100 (11.0%) 808 (89.0%)	41 (31.8%) 88 (68.2%)	59 (7.6%) 720(92.4%)	X²=66.19, *p* = 0.001 X²=66.19, *p* = 0.001

### Operations, laboratory values, and clinical course

Laboratory values were abnormal in *n* = 75 cases (8.3%) preoperatively and in *n* = 129 cases (14.2%) postoperatively, it was notable that, in another *n* = 25 cases (2.8%), no postoperative laboratory control was conducted, these were considered normal for further analyses.

Of the 129 (14.2%) cases with abnormal postoperative laboratory values, 101 (11.1%) had clinically significant increases in leukocytes and/or CRP, and 8 (0.9%) had clinically abnormal bilirubin levels. For the other three patients, clinically significant findings occurred in *n* = 1 (0.1%) for hemoglobin, *n* = 5 (0.6%) for creatinine, and *n* = 3 (0.3%) for potassium. Two or more clinically abnormal parameters were present in *n* = 4 (0.4%) (Table 1).

As a consequence of these laboratory values, further procedures were performed in *n* = 48 patients (5.3%), with the most common being an extended hospitalization for antibiotic therapy (*n* = 27, 3.0%), a laboratory control on the 3rd postoperative day without therapeutic interventions (*n* = 7, 0.8%), an outpatient laboratory control (*n* = 6, 0.7%), or a therapeutic intervention (*n* = 4, 0.4%). Rarely, potassium substitution (*n* = 2, 0.2%), outpatient antibiotic administration (*n* = 1, 0.1%), and a readmission after discharge (*n* = 1, 0.1%) were performed. No re-operation was required for any patient.

In 41 of these 48 cases the hospitalization was extended (> 2 days).

In the other group of patients with normal laboratory values, there were no consequences, but a total of 60 patients (6.3%) had an extended hospitalisation (> 2 days). The reasons for the extended postoperative hospitalisation were: postoperative pain (*n* = 5, 0.6%), wound healing disorder (*n* = 4, 0.5%), elevated infection markers less than the values in our reference range (*n* = 4, 0.5%), and postoperative bleeding (*n* = 3, 0.4%). Less frequently, drainage, defecation disorders, and coagulation disorders occurred with *n* = 1 (0.1%) each, while further diagnostics were mentioned in 7 cases (0.9%). In most cases, however (*N* = 33), no reason was recorded (Table 2).

**Fig. 2:**
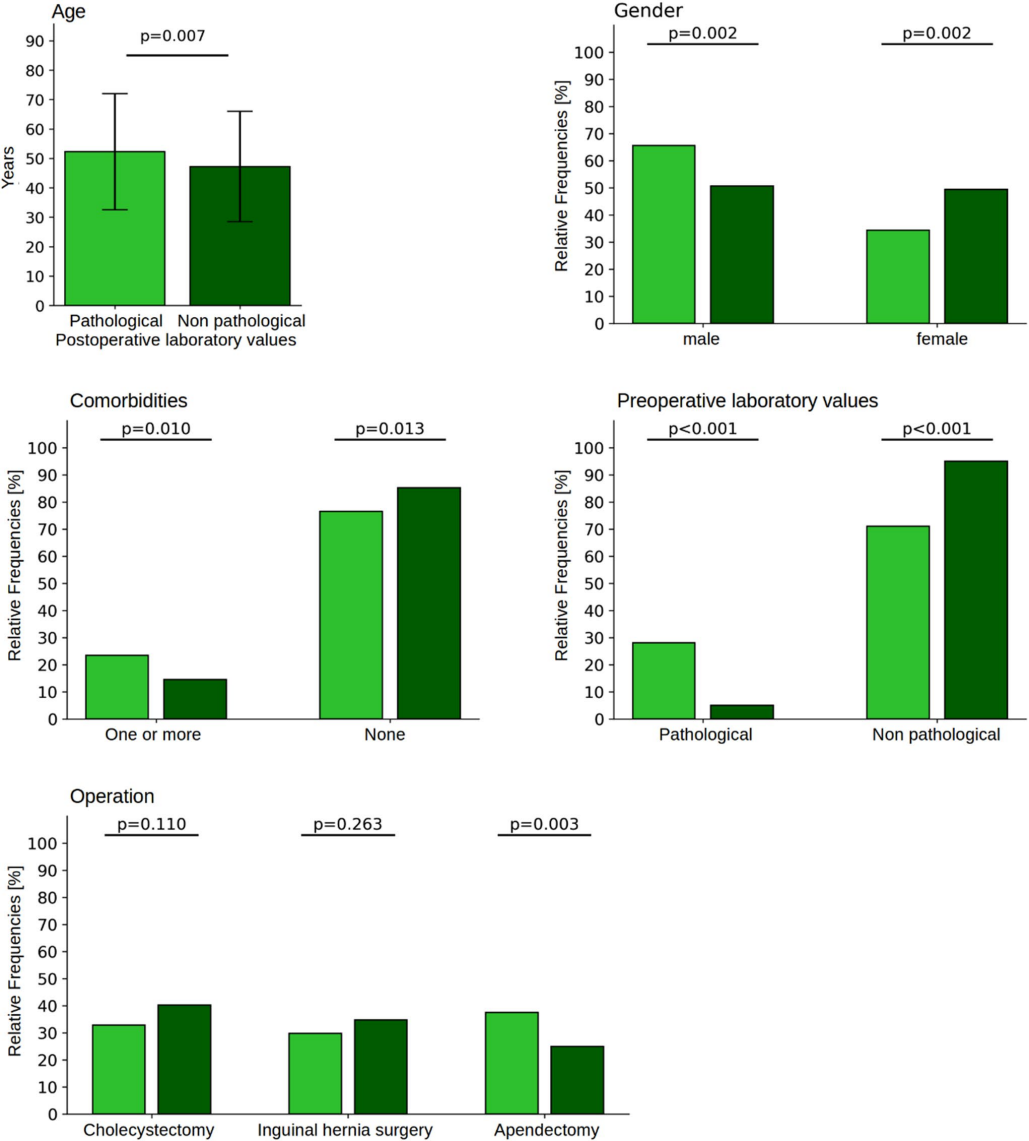
Difference between patients with abnormal (light green bars) and normal (dark green bars) postoperative laboratory values concerning the variables age, gender, comorbidities, findings of the preoperative laboratory, and type of surgery (OP)

### Analysis of preoperative data

To investigate the question of whether there might be specific target groups with known factors in which abnormal postoperative laboratory values occur more frequently, individual factors were examined for their predictive power. The results are shown in Table 1; Fig. 2, where patients with abnormal laboratory values are represented by light green bars and patients with normal laboratory values by dark green bars. As the results show, patients with abnormal laboratory values were significantly older than those with normal values (52.3 vs. 47.2 years, *p* = 0.003), had more men than women represented (about 15% points more men and correspondingly 15% points fewer women, *p* = 0.002), had more comorbidities (8.9% points more, *p* = 0.010), and had nearly six times as many abnormal preoperative laboratory findings (*p* < 0.001).

In relation to the forthcoming surgical procedure, significant discrepancies are observed. While there were only minor and non-significant differences (*p* = 0.236 and *p* = 0.110) among patients with inguinal hernia operations and cholecystectomies, there were significantly more patients with abnormal postoperative laboratory values in appendectomies (in 37.5% instead of 25% of patients, *p* = 0.003).

Overall, the data suggest that a population of typically older male patients with more comorbidities and abnormal preoperative laboratory values undergoing appendectomy tends to have more abnormal postoperative values.

### Analysis of postoperative laboratory values and consequences

As mentioned above, a total of 129 out of 908 patients (14.1%) exhibited abnormal laboratory values postoperatively. This was primarily due to increased inflammation parameters such as leukocytes and CRP levels in 101 of the 129 affected patients (78.1%), while values for bilirubin, creatinine, potassium, and hemoglobin were less frequently abnormal. All these frequencies significantly differed from those in the group of patients classified as having normal postoperative laboratory values. However, the distribution of abnormal values indicates that the only laboratory value that frequently and statistically reliably occurs is the elevation of inflammation parameters leukocytes/CRP.

### Data for cost-benefit analysis

**Fig. 3:**
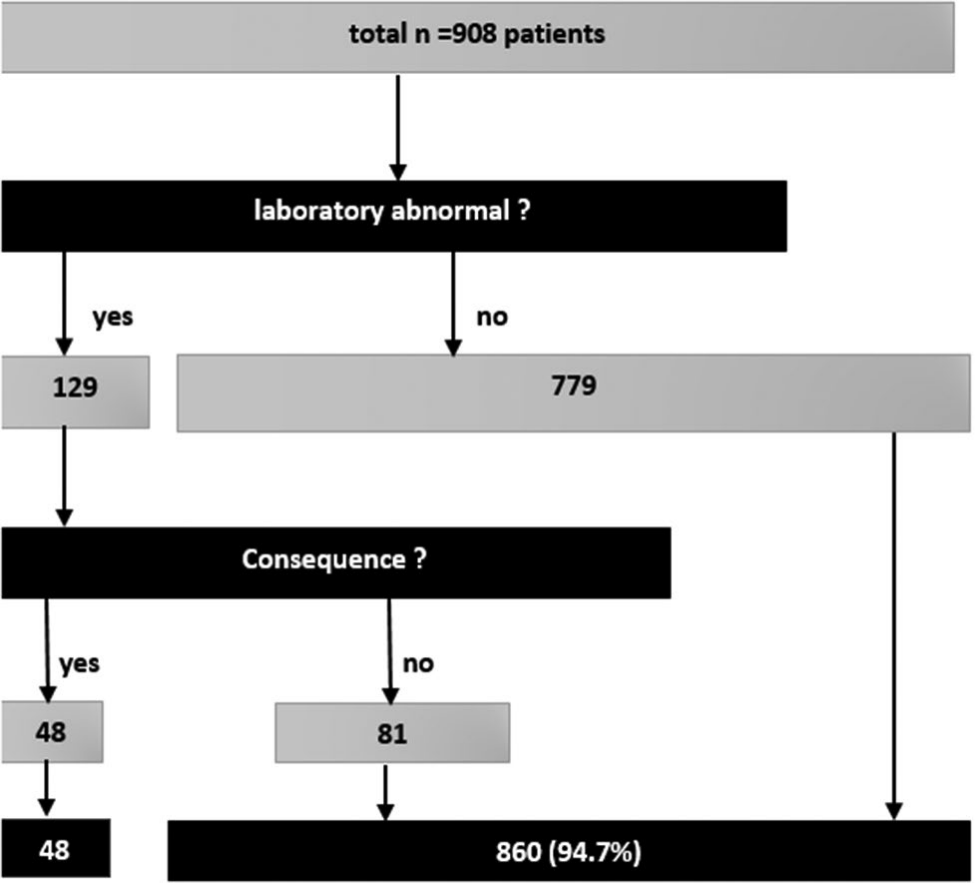
Flow diagram for the distribution of the 908 patients according to the questions of how many cases the postoperative laboratory findings were abnormal or not and how many Positive findings led to consequences. The nember *N* = 48 out of 908 patients positive findings led to consequences. The number *N* = 48 out of 908 patients corresponds to 5.3%

After the last analyses showed that there was no significant correlation between laboratory findings and other abnormalities noted in patient records, the cost-benefit analysis focuses on how much was gained from postoperative laboratory findings. Out of 908 laboratory findings, 129 were positive, of which more than 75% (101 of 129) were due to elevated inflammatory parameters (leukocytes and CRP). Of these 129 cases, only 48 cases (37.5%) had any consequences drawn (Fig. 3).

These 48 cases, from which positive consequences were drawn from the laboratory controls, correspond to 5.3% of the total patient population. If we further divide according to the reason for the surgery, it shows that in appendectomy, there were 28 out of 215 (13.0%), but in cholecystectomy only 15 out of 341 (4.4%) and in inguinal hernia surgeries 5 out of 302 (1.7%).

From these 48 cases, 41 resulted in an extended hospitalisation, with 27 cases due to inpatient antibiotic administration, 7 more cases due to a laboratory control on the third postoperative day, as well as in 4 cases due to a therapeutic intervention and in one case related to a readmission after discharge. In 2 other cases, the extension of hospitalisation was due to intravenous administration of potassium preparations.

### Consequences as a distinguishing point

The above analyses referred to the distinction between abnormal vs. normal laboratory findings as the central grouping variable. However, since not the findings themselves represent the cost-relevant criterion, but rather the consequences, all correlation analyses were conducted again with the question of whether consequences were realized (or not) as a distinguishing variable. The results can be found in Table 2. Although the distinction is.

weaker than in the previously conducted analysis based on laboratory findings, the results point to the same patient group: male patients with comorbidities, abnormal preoperative laboratory values, who underwent an appendectomy rather than an inguinal hernia surgery or a cholecystectomy.

In both analysis groups, with abnormal laboratory values and with consequences drawn,

significant results were obtained. Although the two analyses do not allow for a clear prediction of target patients (with abnormal laboratory values / with consequences drawn) based on factors related to demographics, clinical history, reason for surgery, and course of surgery, they still indicate possibilities for reducing laboratory costs.

**Table 2:** Descriptive results of the study population as a whole (*N* = 908 patients), as well as divided for patients for whom consequences were drawn (*N* = 48) and for whom no consequences were drawn (*N* = 860). The statistical comparison provides the parameters of the t-test (parameter age) and the Chi-squared test (all other parameters). For the parameter age, Descriptive measures are given as m = mean ± sd = standard deviation, for all others in N = absolute frequencies (and relative frequencies in percent)

Consequence					
	Measure	All patients	Yes	No	Stat. comparsion
Number	N(P%)	908 (100%)	48 (100%)	860 (100%)	
Age	M ± SD	47.9 ± 18.98	53.0 ± 22.46	47.6 ± 18.74	T = 1.62, *p* = 0.112
Gender	Male Female	479 (52.8%) 429 (47.2%)	32 (66.7%) 16 (33.3%)	447 (52.0%) 413 (48.0%)	X²=3.94, *p* = 0.047 X²=3.94, *p* = 0.047
Preop. lab.	Abnormal normal	75 (8.3%) 832 (91.6%)	25 (52.1%) 22 (45.8%)	50 (5.8%) 810 (94.2%)	X²=128.44, *p* = 0.001 X²=138.59, *p* = 0.001
Operation	Cholecystectomy Inguinal hernia repair Appendectomy	356 (39.2%) 309 (34.0%) 243 (26.8%)	15 (31.2%) 5 (10.4%) 28 (58.3%)	341 (39.7%) 304 (35.3%) 215 (25.0%)	X²=1.35, *p* = 0.246 X²=12.59, *p* = 0.001 X²=25.77, *p* = 0.001
Postoperative laboratory values	Abnormal normal no check	129 (14.2%) 754 (83.0%) 25 (2.8%)	48 (100%) 0 (0.0%) 0 (0.0%)	81 (9.4%) 754 (87.7%) 25 (2.9%)	X²=306.04, *p* = 0.001 X²=248.13, *p* = 0.001 X²=1.43, *p* = 0.231
Abnormal laboratory parameter	Leucocytes or CRP Hemoglobin Creatinine Bilirubin Potassium more than one	101 (11.1%) 1 (0.1%) 5 (0.6%) 8 (0.9%) 3 (0.3%) 4 (0.4%)	35 (72.9%) 0 (0.0%) 1 (2.1%) 5 (10.4%) 2 (4.2%) 3 (6.2%)	66 (7.7%) 1 (0.1%) 4 (0.5%) 3 (0.3%) 1 (0.1%) 1 (0.1%)	X²=195.74, *p* = 0.001 X²=0.06, *p* = 0.813 X²=2.17, *p* = 0.140 X²=52.77, *p* = 0.001 X²=22.65, *p* = 0.001 X²=39.00, *p* = 0.001
Consequence	yes no	48 (5.3%) 858 (94.5%)	48 (5.3%) 0 (0.0%)	0 (0.0%) 858 (94.5%)	X²=908.00, *p* = 0.001 X²=869.65, *p* = 0.001
Procedure	Inpatient antibiotic ther. labor check on day 3 therapeutic intervention hospital readmission potassium replacement outpatient lab. check outpatient antibiotic therapy	27 (3.0%) 7 (0.8%) 4 (0.4%) 1 (0.1%) 2 (0.2%) 6 (0.7%) 1 (0.1%)	27 (56.2%) 7 (14.6%) 4 (8.3%) 1 (2.1%) 2 (4.2%) 6 (12.5%) 1 (2.1%)	0 (0.0%) 0 (0.0%) 0 (0.0%) 0 (0.0%) 0 (0.0%) 0 (0.0%) 0 (0.0%)	X²=498.58, *p* = 0.001 X²=126.39, *p* = 0.001 X²=71.98, *p* = 0.001 X²=17.94, *p* = 0.001 X²=35.91, *p* = 0.001 X²=108.22, *p* = 0.001 X²=17.94, *p* = 0.001
Postoperative complications	postoperative bleeding postoperative pain drain elevated infection markers wound infection defecation disorders coagulation disorders marcumar therapy medical consultation unknown	3 (0.3%) 5 (0.6%) 1 (0.1%) 4 (0.4%) 4 (0.4%) 1 (0.1%) 1 (0.1%) 1 (0.1%) 7 (0.8%) 36 (4.0%)	0 (0.0%) 0 (0.0%) 0 (0.0%) 0 (0.0%) 0 (0.0%) 0 (0.0%) 0 (0.0%) 0 (0.0%) 0 (0.0%) 0 (0.0%)	3 (0.3%) 5 (0.6%) 1 (0.1%) 4 (0.5%) 5 (0.6%) 1 (0.1%) 1 (0.1%) 1 (0.1%) 7 (0.8%) 36 (4.2%)	X²=0.17, *p* = 0.682 X²=0.28, *p* = 0.596 X²=0.06, *p* = 0.813 X²=0.22, *p* = 0.636 X²=0.22, *p* = 0.636 X²=0.06, *p* = 0.813 X²=0.06, *p* = 0.813 X²=0.06, *p* = 0.813 X²=0.39, *p* = 0.530 X²=2.09, *p* = 0.048
Duration of hospitalisation ( normal ≤ 2 days)	abnormal normal	100 (11.0%) 808 (89.0%)	37 (77.1%) 11 (22.9%)	63 (7.3%) 797 (92.7%)	X²=225.74, *p* = 0.001 X²=225.74, *p* = 0.001

### Predictive models for consequences and length of hospitalisation

After the above analyses showed that postoperative laboratory values are an important factor in decisions, but that other (preoperative) factors are related to whether the postoperative laboratory is abnormal, it should finally be clarified using logistic regression which factors most strongly predict the questions of consequences drawn (yes/no) and an extended length of hospitalisatio (yes/no). The strength of the Wald statistic (z) indicates their influence.

Table 3 shows the results. For the decision regarding whether consequences occur or not, the laboratory findings before (z = 7.07) and after the operation (z = 7.05) are equally decisive, while age plays a lesser but still significant role (z = 3.07). For the decision regarding an extended hospitalisation, the laboratory findings before the operation (z = 4.66) play the most important role just ahead of age (z = 3.99), while the pure laboratory finding after the operation with a z = 1.96 is just significant, but comes in fourth place behind the type of surgery (z = 2.45).

**Table 3:** Results of the two logistic regressions for the target parameters consequences drawn (yes/no) and extended hospitalisation (yes/no) depending on the predictors OP (type), age, gender, preoperative laboratory, laboratory after the OP. B = regression coefficient, se = standard error of the coefficient, 95%ci = confidence interval for the coefficient with lower and upper limits, z = wald statistic of the regression analysis and p = probability of error

target parameters	predictors	B	SE	[95% CI]	z	*p*
**consequences**	OP	0,05	0,29	[-0.52– 0.62]	0,18	0,857
	age	0,03	0,01	[0.01– 0.05]	3,07	0,002
	gender	-0,22	0,39	[-0.98– 0.54]	-0,57	0,568
	preop. laboratory	3,34	0,47	[2.42– 4.27]	7,07	< 0.001
	postop. laboratory	1,28	0,18	[0.92– 1.63]	7,05	< 0.001
**extended hospitalisation**	OP	0,43	0,17	[0.09– 0.76]	2,46	0,014
	age	0,03	0,01	[0.01– 0.04]	3,99	< 0.001
	gender	-0,15	0,24	[-0.63– 0.33]	-0,63	0,532
	preop. laboratory	1,50	0,32	[0.87– 2.13]	4,66	< 0.001
	postop. laboratory	0,31	0,16	[0.00– 0.62]	1,95	0,052

## Discussion

The presented study analyses the value of routine postoperative blood sample analysis for standard minor procedures in general surgery. While the motivation for routine testing may have historical and forensic roots, the focus today is more and more shifted towards the economical impact and patient comfort [16–18]. Regarding the increasing trend towards ambulatory care for minor general surgery the necessity and relevance should critically be evaluated [19, 20].

As decribed above our patient collective consisted of groin hernia repairs, cholecystectomies and appendectomies. In this rather large two-year single-centre collective only the appendectomy cases somewhat “justified” the postoperative blood sampling.

For groin hernia patients it could clearly be demonstrated that no additional information or consequence was generated by POD-1 blood value analysis. It may therefore be postulated that - exept for special cases with explicit demand by the operating surgeon– POD-1 blood sampling should be omitted from the clinical routine.

The same is obvious for elective cholecystectomy cases in the absence of severe inflammation. If the intraoperative course demands further analysis (f.i. bleeding, difficult anatomy, bile leackage,…) the operating surgeon may explicitely demand POD-1 blood sampling. For the vast majority of uncomplicated cholecystectomy cases it should be discarded from routine practice [21–23].

For appendectomy cases the picture is more heterogeneous as POD-1 blood values did have an influence on the further clinical course in 13% of the cases. However, our further analysis also demonstrated that preoperative blood values and the clinical patient status generated almost identical consequences. In cases with severe intraoperative inflammation, though, postoperative blood sampling can serve to monitor the success of antibiotic treatment. POD-1 blood sampling, however, is normally too early to gain useful information. For appendectomies it may therefore be stated that routine POD-1 blood sampling is of little use and may be omitted. For patients with severe intraoperative inflammation / surgical problems / postoperative antibiotic treatment at least one postoperative blood value analysis should be performed before discharge [24].

Annually, substantial numbers of surgeries are performed in Germany: 175 000 cholecystectomies [25], 275 000 inguinal hernia repairs [26] and 135 000 appendectomies [27], totaling around 585 000 surgeries per year. Consequently, the costs associated with routine postoperative laboratorory examination can be significantly reduced.

The most common consequence is a prolonged hospital stay due to antibiotic therapy, while the significant consequences, such as re-operation (not a single patient) or therapeutic interventions (4 patients), were notably less frequent. It is also doubtful whether the laboratory infection values would have spontaneously decreased without antibiotic therapy; prospective studies are required to answer this question.

As demonstrated in our study the intensity of intraoperatively detected inflammation combined with the technical complexity of the procedure and the inherent individual patient risk factors (age / co-morbidities) should trigger application of POD-1 blood sampling. For patients receiving antibiotic treatment postoperatively POD-2 blood value analysis may be more useful.

For the vast majority of patients in our study population–which is somewhat representative for the real-world circumstances in German hospitals- postoperative blood value analysis could be omitted completely without negative consequences.

## Conclusion

The presented data strongly supports strictly limited application of routine POD-1 blood sample analysis for minor general surgery procedures. Thereby substantial cost savings paired with reduced patients’ distress could be generated.

## Data Availability

No datasets were generated or analysed during the current study.
